# Identification of Genetic Signature Associated With Aging in Pulmonary Fibrosis

**DOI:** 10.3389/fmed.2021.744239

**Published:** 2021-10-20

**Authors:** Yanjiao Lu, Jinkun Chen, Shanshan Wang, Zhen Tian, Yan Fan, Meijia Wang, Jianping Zhao, Kun Tang, Jungang Xie

**Affiliations:** ^1^Department of Respiratory and Critical Care Medicine, National Clinical Research Center of Respiratory Disease, Key Laboratory of Pulmonary Diseases of Health Ministry, Tongji Hospital, Tongji Medical College, Huazhong University of Science and Technology, Wuhan, China; ^2^Department of Science, Western University, London, ON, Canada; ^3^Department of Pulmonary and Critical Care Medicine, The First Affiliated Hospital of Sun Yat-sen University, Guangzhou, China; ^4^Institute of Respiratory Diseases of Sun Yat-sen University, Guangzhou, China

**Keywords:** aging, pulmonary fibrosis, genetic signature, idiopathic pulmonary fibrosis, glucocorticoids

## Abstract

**Background:** Aging is a strong risk factor and an independent prognostic factor in idiopathic pulmonary fibrosis (IPF). In this study, we aimed to conduct a comprehensive analysis based on gene expression profiles for the role of aging in pulmonary fibrosis.

**Method:** Four datasets (GSE21411, GSE24206, GSE47460, and GSE101286) for patients with clinical IPF and one dataset for bleomycin (BLM)-induced pulmonary fibrosis (BIPF) mouse model (GSE123293) were obtained from Gene Expression Omnibus (GEO). According to different age ranges, both patients with IPF and BIPF mice were divided into young and aged groups. The differently expressed genes (DEGs) were systemically analyzed using Gene Ontology (GO) functional, Kyoto Encyclopedia of Genes and Genomes (KEGG), and hub genes analysis. Finally, we verified the role of age and core genes associated with age *in vivo*.

**Results:** *Via* the expression profile comparisons of aged and young patients with IPF, we identified 108 aging-associated DEGs, with 21 upregulated and 87 downregulated. The DEGs were associated with “response to glucocorticoid,” “response to corticosteroid,” and “rhythmic process” in GO biological process (BP). For KEGG analysis, the top three significantly enriched KEGG pathways of the DEGs included “IL-17 signaling pathway,” “Mineral absorption,” and “HIF-1-signaling pathway.” Through the comparisons of aged and young BIPF mice, a total number of 778 aging-associated DEGs were identified, with 453 genes increased and 325 genes decreased. For GO and KEGG analysis, the DEGs were enriched in extracellular matrix (ECM) and collagen metabolism. The common DEGs of patients with IPF and BIPF mice were enriched in the BP category, including “induction of bacterial agglutination,” “hyaluronan biosynthetic process,” and “positive regulation of heterotypic cell-cell adhesion.” We confirmed that aged BIPF mice developed more serious pulmonary fibrosis. Finally, the four aging-associated core genes (*Slc2a3, Fga, Hp*, and *Thbs1*) were verified *in vivo*.

**Conclusion:** This study provides new insights into the impact of aging on pulmonary fibrosis. We also identified four aging-associated core genes (*Slc2a3, Fga, Hp*, and *Thbs1*) related to the development of pulmonary fibrosis.

## Introduction

The process of aging is featured by gradual functional impairments of tissue and organisms of the body, leading to reduced resilience to environmental damages and increased risks of occurrence of diseases and death (1). Idiopathic pulmonary fibrosis (IPF) is an age-associated chronic lethal disease caused by an unknown reason (2). It is commonly diagnosed in patients over 50 years old, and the incidence of this disease significantly increases in the population older than 50 years old. (3). For this reason, better understandings of the pathophysiology of IPF and how the process of aging is related to the incidence of IPF are necessary for the development of new therapies for clinical treatments of this disease.

Despite the repeated proposals of the association between aging and IPF, the mechanism underlying this association is still unclear (4, 5). Lopez-Otin et al. enumerate nine tentative aging hallmarks: genomic instability, telomere attrition, epigenetic alterations, loss of proteostasis, deregulated nutrient sensing, mitochondrial dysfunction, cellular senescence, stem cell exhaustion, and altered intercellular communication (6). Meanwhile, heterozygous mutations in four telomere-related genes are also found to associate with the development of pulmonary fibrosis (7). Moreover, biomarkers of aging, including p21 and p16, are also found significantly upregulated in both epithelial cells and fibroblasts in lung tissue of patients with IPF as compared with that of healthy people (8). In animal studies of IPF using the bleomycin (BLM)-induced pulmonary fibrosis (BIPF), aged BIPF mice failed to resolve fibrosis as compared with young mice (9). However, despite the wide-recognized association between aging and IPF, its potential mechanism is unclear (10).

In this study, to investigate the mechanisms underlying the association between pulmonary fibrosis and aging, using gene expression profiles of both patients with IPF and BIPF mice, we comprehensively analyzed the effects of aging in the development of pulmonary fibrosis and verified our findings in *in vivo* studies.

## Method

### Microarray Data Acquisition and Process

To identify the genes associated with aging in patients with IPF, four microarray datasets (GSE21411, GSE24206, GSE47460, and GSE101286) were downloaded from Gene Expression Omnibus (GEO, http://www.ncbi.nlm.nih.gov/geo) (11–14). IPF was diagnosed according to the ATS/ERS criteria published (15, 16). Young patients with IPF were defined as age ≤ 55 years old, and aged patients with IPF were defined as age ≥70 years old. Gender- and age-matched healthy controls were gotten from GSE47460. In order to explore the genes associated with age in BIPF mice, we downloaded one microarray dataset (GSE123293) from GEO (17). In this dataset, mice were divided into young (2–4 months old) and aged (18–20 months old) groups. Gender- and age-matched mouse controls were from GSE123293. Detailed information of GSE datasets was summarized in Table 1. Preprocessed expression matrix and probe annotation files of the four GSE datasets were obtained from the GEO repository. Probe annotations and the corresponding expression profiles from different datasets were mapped into a common gene expression list according to the method described by Shi et al. (18). Probes obtained from different datasets were listed using official gene symbols. Multiple expression results of the certain gene were replaced by the median value of the expression results (19, 20). Log2 fold-change (FC) of all expression results was normalized using the Cross-platform Normalization (xpn) method by platforms.

**Table 1:** Characteristics of studies included.

**Dataset**	**Platform**	**Type of sample**	**Sample (Young group)**	**Sample (Aged group)**	**Reference**
GSE21411	GPL570	IPF lung tissue	2	0	(11)
GSE24206	GPL570	IPF lung tissue	1	1	(12)
GSE47460	GPL6480	IPF lung tissue	16	29	(13)
	GPL14550				
		Healthy lung tissue	23	23	
GSE101286	GPL6947	IPF lung tissue	0	3	(14)
GSE123293	GPL16570	BIPF lung tissue	3	3	(17)
		Healthy lung tissue	3	3	

*IPF, idiopathic pulmonary fibrosis; BIPF, bleomycin induced pulmonary fibrosis*.

Differently expressed genes (DEGs) analysis, Gene Ontology (GO) functional analysis, and Kyoto Encyclopedia of Genes and Genomes (KEGG) analysis were performed.

To identify DEGs between young and aged groups, the limma R package (http://www.bioconductor.org/packages) was used to perform the negative binomial distribution method according to the absolute value of FC (>1.5) and false discovery rate (FDR) <0.05. According to the hypergeometric distribution algorithm, pathway enrichment analyses of the GO biological process (BP), molecular function (MF), and cell component (CC) were carried out by the “cluster-profler” R package. The same method was applied for the KEGG analysis. The cutoff value was *p* < 0.05. The Cytoscape (version 3.8.0) was used to select hub or core genes.

### Animal

Specific pathogen-free (SPF) C57 BL/6N male mice (young mice, 2–4 months old; aged mice, 18–20 months old) were purchased from the HFK Bioscience company (Beijing, China). All animals were kept under the SPF environment at Tongji Medical College. All procedures for animal experiments were approved by the Animal Care and Use Committee of Tongji Hospital.

The animal model of pulmonary fibrosis was established by single intratracheal administration of BLM (2 mg/kg, MCE, USA) or normal saline (NS) as control. Mice were grouped as follows: NS (young mice intratracheal with NS), aged NS (aged mice intratracheal with NS), BLM (young mice intratracheal with BLM), and aged BLM (aged mice intratracheal with BLM). Mice were sacrificed 21 days after the establishment of the BIPF mice model. Bronchoalveolar lavage fluid (BALF) was carried out. Samples of lung tissue were collected. The left lung was paraffin embedded for hematoxylin-eosin (HE) staining and masson trichrome (MT) staining (21). Lung hydroxyproline contents were measured by hydroxyproline colorimetric assay (Biovision, Milpitas, USA).

### Quantitative Real-Time Polymerase Chain Reaction (RT-PCR)

Briefly, the total RNA of the lung tissue was extracted by RNAiso plus kit (TaKaRa) and reversely transcripted to cDNA. RT-PCR was carried out using SYBR Premix Ex Taq (Tarkara, Shiga, Japan). The relative mRNA expression was calculated using the 2^−Δ*ΔCT*^ method normalized to the level of β-actin. The primers of four core genes are listed in Supplementary Table 1.

### Statistical Analysis

Data analysis was carried out by Rstudio (version 3.6.2) and GraphPad Prism 7. The microarray data were analyzed by different R packages. Results were expressed as means ± SEM. A two-way analysis of variance (ANOVA) followed by Tukey's multiple comparison post-test was used. *p* < 0.05 was considered as statistically significant.

## Results

### DEGs Associated With Age in IPF

**Figure 1:**
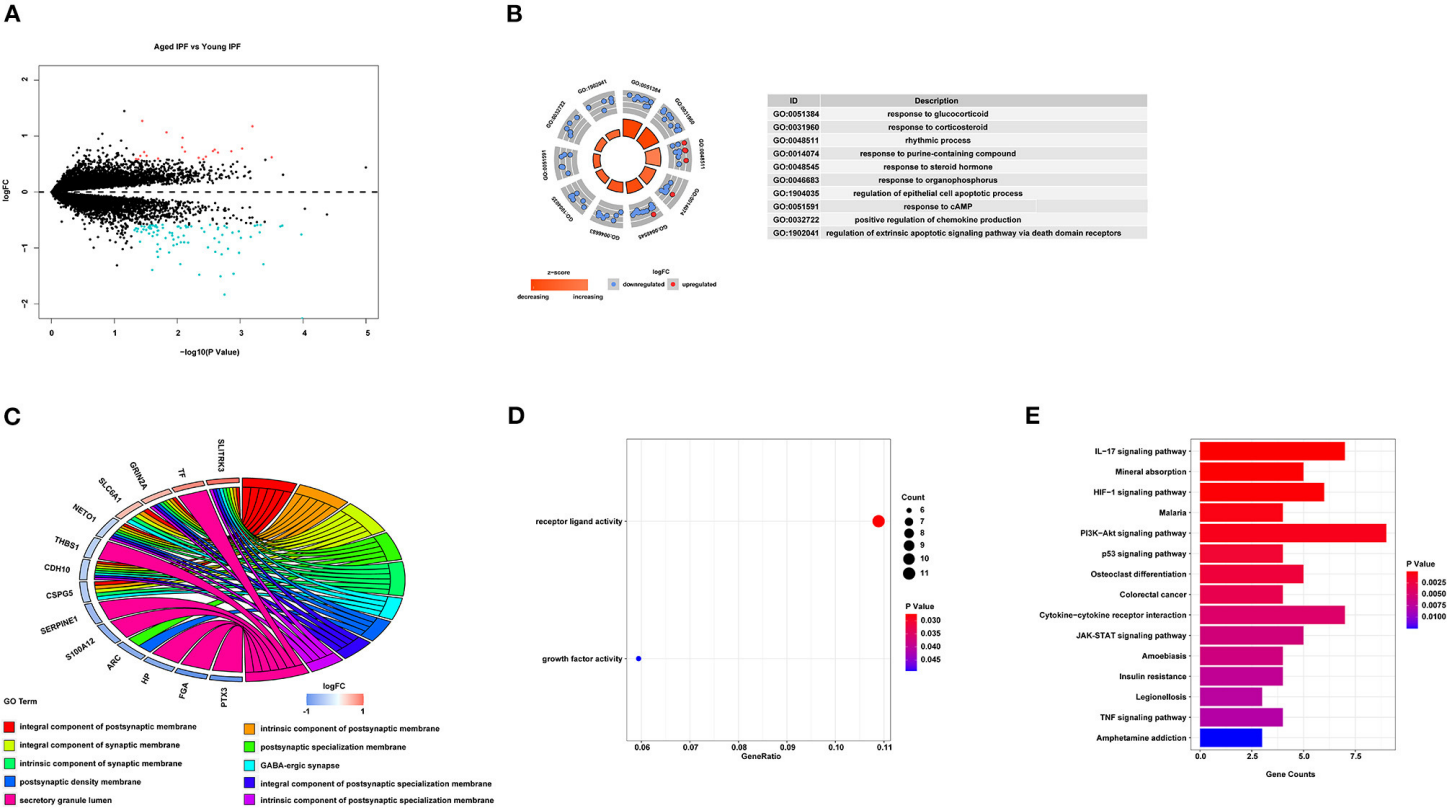
DEGs associated with age in IPF. **(A)** Volcano-plot of the 108 age-associated DEGs in patients with IPF. Pink circle: upregulated genes with fold change over 1.5; blue circles: downregulated genes with a fold change over 1.5. **(B)** Top 10 GO biological processes analysis of the 108 age-associated DEGs. Outer gray circle: a scatter plot for each term of the logFC of the assigned genes; red circles: upregulation genes; blue circles: downregulation genes. **(C)** Top 10 GO cellular components analysis of the 108 age-associated DEGs. **(D)** All GO molecular functions analysis of the 108 age-associated DEGs. **(E)** Top 15 significantly enriched KEGG pathways. DEGs, differently expressed genes; IPF, idiopathic pulmonary fibrosis; GO, gene ontology; FC, fold change; KEGG, Kyoto Encyclopedia of Genes and Genomes.

To determine the core gene profiles related to age in patients with IPF, four microarray datasets were obtained from GEO. Information of the included studies is listed in Table 1; Supplementary Table 2. According to the age criterion, this study included 19 young patients with IPF and 33 aged patients with IPF. Gene expression profiles were compared between the two groups, generating 108 DEGs, with 21 upregulated and 87 downregulated (Figure 1A). For further understanding of the function related to these 108 DEGs, GO enrichment analysis, including BP, MF, and CC, was performed. In BP analysis, the DEGs were found to associate with “response to glucocorticoid,” “response to corticosteroid,” and “rhythmic process” (Figure 1B). In the CC category, the DEGs were related to the “integral component of postsynaptic membrane,” “intrinsic component of postsynaptic membrane,” and “integral component of synaptic membrane” (Figure 1C). Moreover, DEGs were enriched in the MF category related to “receptor ligand activity” and “growth factor activity” (Figure 1D). For KEGG pathway enrichment analysis, the top three significant KEGG pathways of the DEGs included “IL-17 signaling pathway,” “Mineral absorption,” and “HIF-1-signaling pathway” (Figure 1E). More information on IPF DEGs analysis was shown in Supplementary File 1. The same biological analysis was performed in human young healthy control (HC) and aged HC (Supplementary File 3; Supplementary Figure 1). Different from young and aged IPF, DEGs of young and aged HC were enriched in inflammation and immune.

### DEGs Associated With Age in BIPF Mice

**Figure 2:**
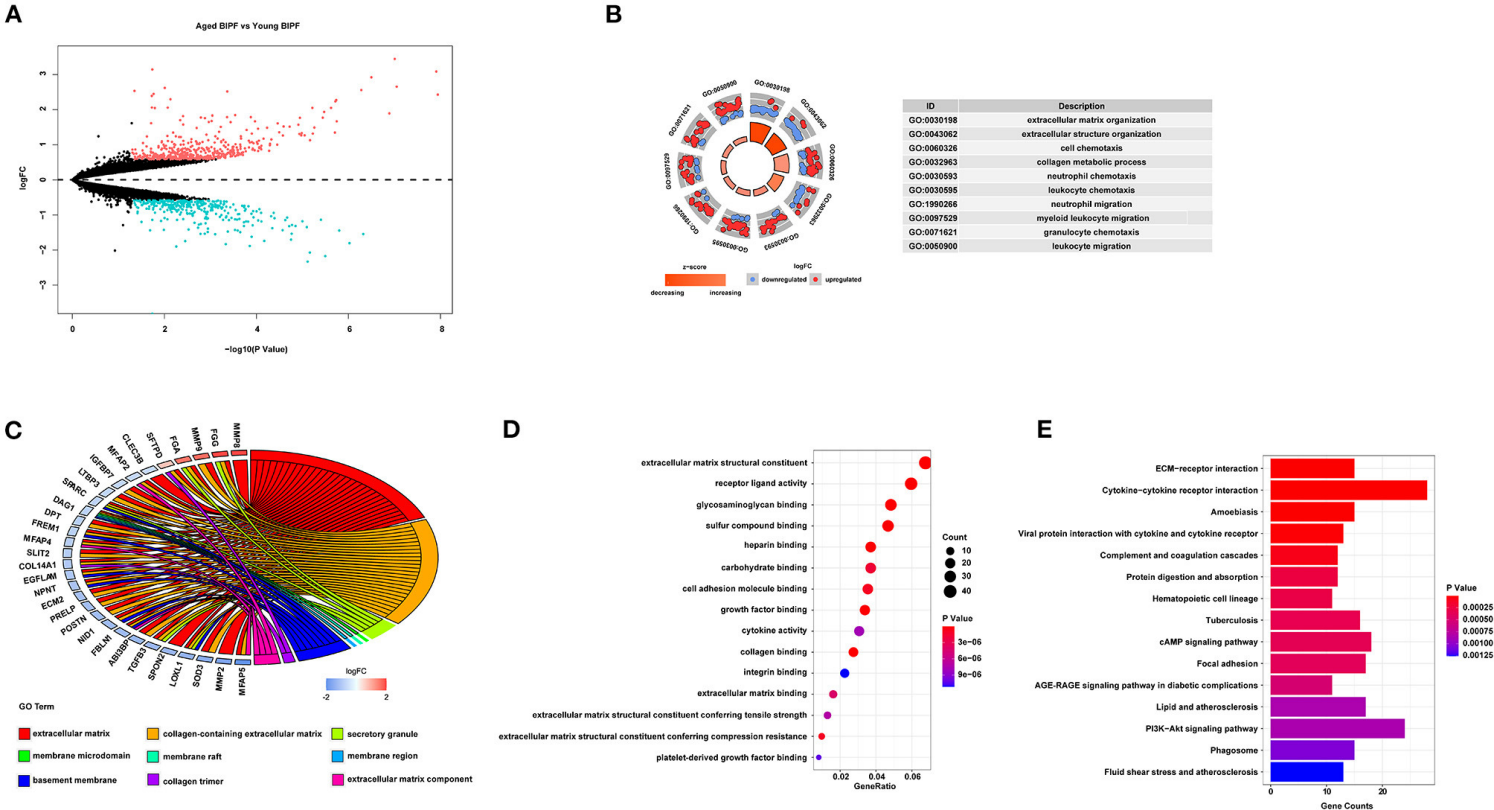
Age-associated DEGs in BIPF mice. **(A)** Volcano plot of the 778 age-associated DEGs. Pink circle: upregulated genes with fold change over 1.5; blue circles: downregulated genes with a fold change over 1.5. **(B)** Top 10 GO biological processes of the 778 age-associated DEGs. Outer gray circle: a scatter plot for each term of the logFC of the assigned genes; red circles: upregulation genes; blue circles: downregulation genes. **(C)** Top nine GO cellular components of the 778 age-associated DEGs. **(D)** Top 15 GO molecular functions of the 778 age-associated DEGs. **(E)** Top 15 significantly enriched KEGG pathways. DEGs, differently expressed genes; BIPF, bleomycin-induced pulmonary fibrosis; GO, gene ontology; FC, fold change; KEGG, Kyoto Encyclopedia of Genes and Genomes.

For further identification of the expression features associated with aging in BIPF mice, we obtained one dataset from GEO and compared the DEGs between aged and young BIPF mice. A total number of 778 DEGs were identified, with 453 genes increased and 325 genes decreased (Figure 2A). Slightly different from the results obtained from patients with IPF, the top five BP terms were enriched in “extracellular matrix organization,” “extracellular structure organization,” “cell chemotaxis,” “collagen metabolic process,” and “neutrophil chemotaxis” (Figure 2B). In the CC category, the DEGs were associated with “extracellular matrix,” “collagen-containing extracellular matrix,” and “extracellular matrix component” (Figure 2C). “Extracellular matrix component,” “glycosaminoglycan binding,” and “collagen binding” were the most important MF terms (Figure 2D). Furthermore, KEGG pathway analysis showed enrichments in “ECM-receptor interaction,” “Cytokine-cytokine receptor interaction,” and “Amoebiasis” (Figure 2E). Detailed information of BIPF DEGs analysis was displayed in Supplementary File 2. The same bioinformatical analysis was conducted in young mouse control (MC) and aged MC (Supplementary File 4; Supplementary Figure 2). Similar to HC age-associated DEGs, inflammation and immune were enriched in DEGs of young and aged MC.

### Common DEGs Associated With Age in Pulmonary Fibrosis

**Figure 3:**
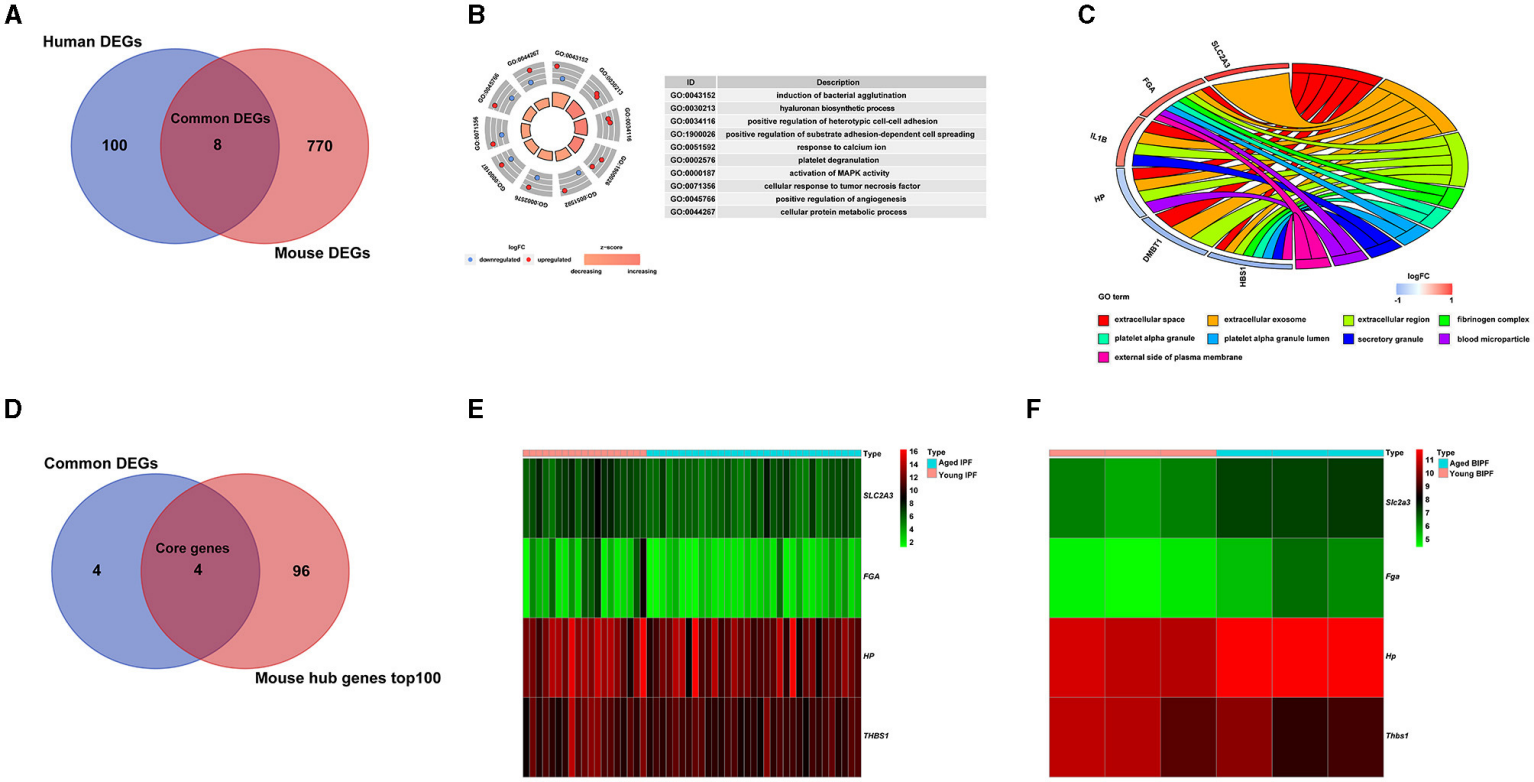
Common age-associated DEGs in pulmonary fibrosis. **(A)** A Venn diagram of DEGs of patients with IPF and BIPF mice. **(B)** Significant enrichments of GO biological processes analysis of the eight common DEGs. **(C)** Significant enrichments of GO cellular components analysis of the eight common DEGs. **(D)** A Venn diagram of core genes extracted by common DEGs analysis and mouse hub genes datasets. **(E)** Heatmap of the four core genes in young and aged IPF. **(F)** Heatmap of the four core genes in young and aged BIPF mice. DEGs, differently expressed genes; IPF, idiopathic pulmonary fibrosis; BIPF, bleomycin-induced pulmonary fibrosis; GO, gene ontology.

To comprehensively explore the effects of aging in pulmonary fibrosis, we combined the results of patients with IPF and BIPF mice. As shown in the Venn diagram, eight common DEGs were found in both aged patients with IPF and aged BIPF mice (Figure 3A). The eight common DEGs were enriched in the BP category related to “induction of bacterial agglutination,” “hyaluronan biosynthetic process,” and “positive regulation of heterotypic cell-cell adhesion” (Figure 3B). In CC analysis, the eight common genes were associated with “extracellular space,” “extracellular exosome,” and “extracellular region” (Figure 3C). Subsequently, to confirm the aging-related core genes, we overlapped the eight common genes with the top 100 core DEGs in BIPF mice. In the results, the four aging-related core genes were identified: solute carrier family 2 member 3 (*Slc2a3)*, fibrinogen alpha chain (Fga), haptoglobin (H*p*), and thrombospondin 1 (*Thbs1*) (Figure 3D). The heatmap showed the four core genes expression in both patients with IPF and BIPF mice (Figures 3E,F). Detailed information on the four core genes is displayed in Table 2. There was no common gene among aged and young IPF DEGs, BIPF DEGs, HC DEGs, and MC DECs (Supplementary Figure 3; Supplementary File 5).

**Table 2:** List of 4 core genes.

**Human**				**Mouse**			
**Gene**	**ID**	**log2 FC**	***P* value**	**Gene**	**ID**	**log2 FC**	***P* value**
*SLC2A3*	6515	−0.801	0.00068	*Slc2a3*	20527	1.14913	4.74E−05
*FGA*	2243	−1.462	0.00017	*Fga*	14161	1.42123	0.0001638
*HP*	3240	−1.162	0.02032	*Hp*	15439	0.86172	0.000122
*THBS1*	7057	−0.611	0.03689	*Thbs1*	21825	−0.94997	0.038588

*Human: aged IPF vs. young IPF; mouse: aged BLM vs. young BLM; ID, entrez ID; FC, fold change*.

### *In vivo* Validations of the Impact of Aging and Four Core Genes

**Figure 4:**
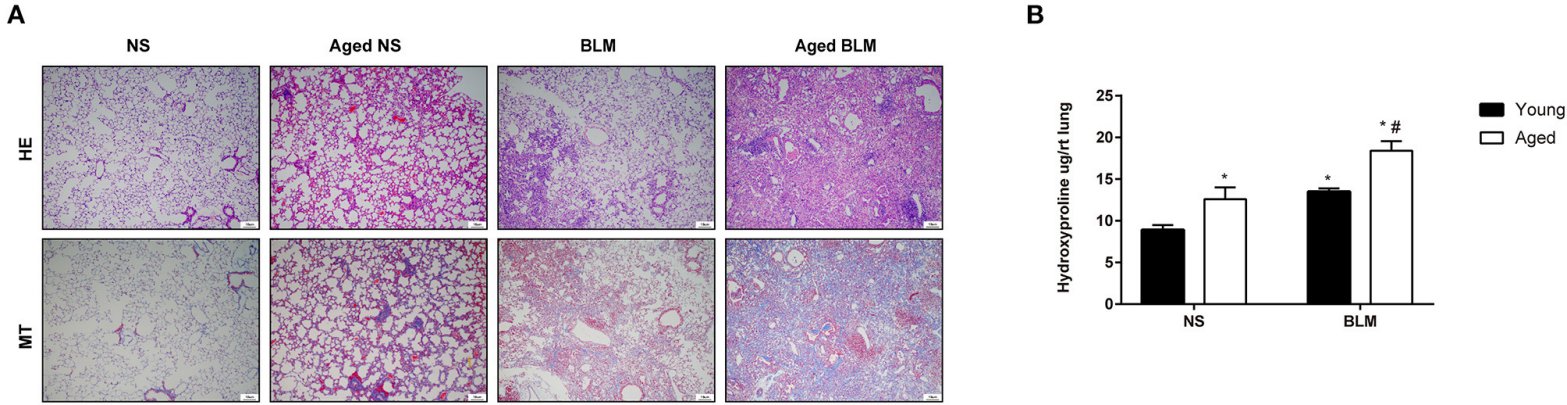
Aging aggravates pulmonary fibrosis *in vivo*. Young C57BL/6N male mice (2–4 months) and aged C57BL/6 male mice (16–18 months) were intratracheally injected with 50 μl of NS or 2-mg/kg BLM. **(A)** Representative HE and MT staining images of lung sections of each group. Magnification X200. Scale bar, 10 um. **(B)** Hydroxyproline measurements. The four groups were NS (young mice intratracheal with NS), aged NS (aged mice intratracheal with NS), BLM (young mice intratracheal with BLM), and aged BLM (aged mice intratracheal with BLM). Values were expressed as mean ± SEM (*n* = 4). *p < 0.05, compared with the young NS group, ^#^*p* < 0.05, compared with the young BLM group. Two-way ANOVA was used. NS, normal saline; BLM, bleomycin; HE, hematoxylin-eosin staining; MT, masson trichrome staining.

**Figure 5:**
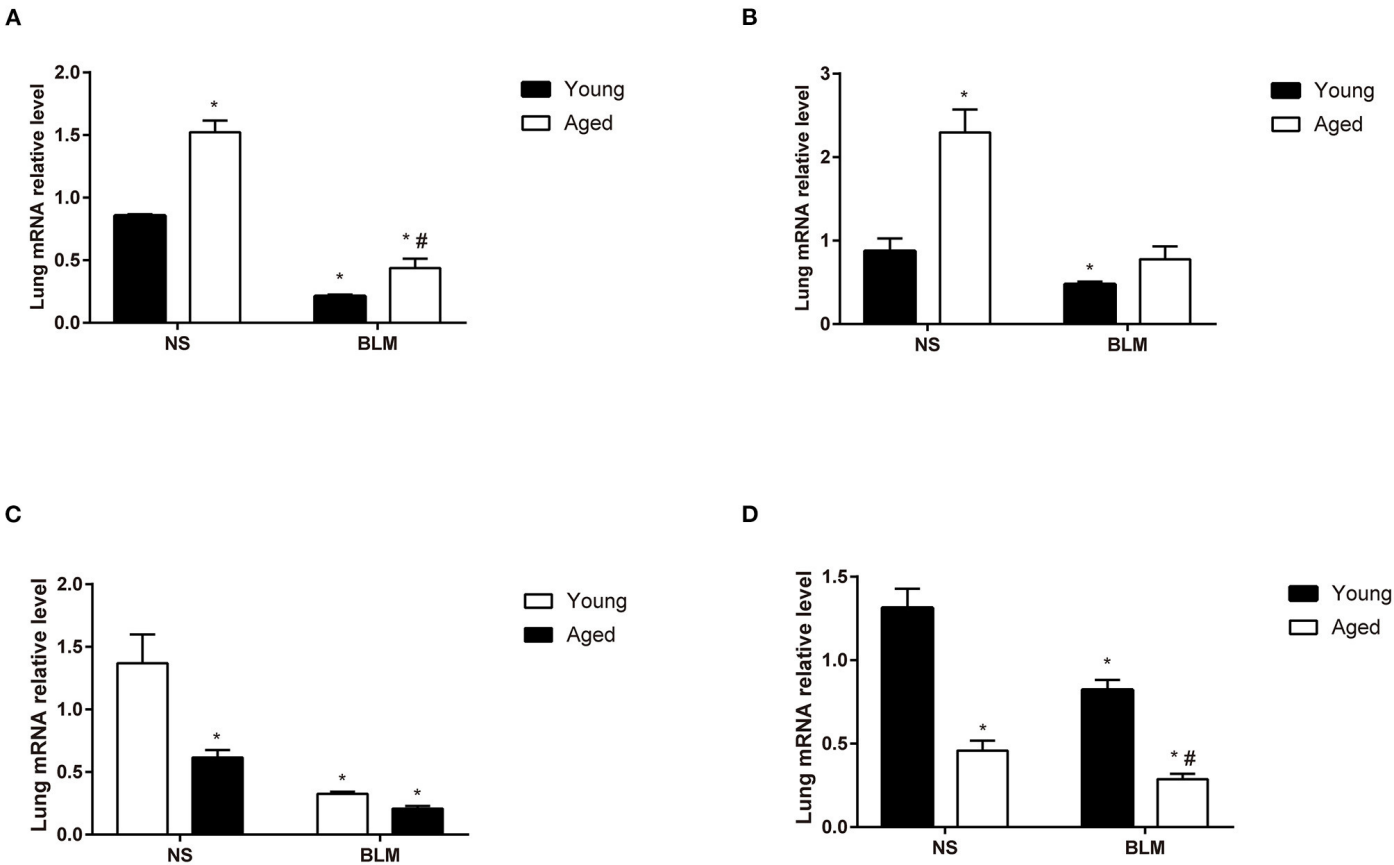
The expression of the four core genes in the lungs of BIPF mice. **(A)** *Slc2a3*, **(B)** *Fga*, **(C)** *Hp*, and **(D)** *Thbs1* in the NS, aged NS, BLM, and aged BLM groups. The four groups were NS (young mice intratracheal with NS), aged NS (aged mice intratracheal with NS), BLM (young mice intratracheal with BLM), and aged BLM (aged mice intratracheal with BLM). Values were expressed as mean ± SEM (*n* = 4), *p < 0.05, compared with the young NS group, ^#^*p* < 0.05, compared with the young BLM group. Two-way ANOVA was used. BIPF, bleomycin-induced pulmonary fibrosis; NS, normal saline; BLM, bleomycin.

Aging is an important risk factor in patients with IPF. In this study, we compared the fibrosis degree between young and aged mice. In histopathological analysis, we found that, with the increase of age, fibrosis appeared in lung tissue spontaneously (Figures 4A,B). Under the stimulation of BLM, more serious pulmonary fibrosis was developed in aged mice. This finding was consistent with the previous study (5). For further confirmation of the expression changes in the four core genes (*Slc2a3, Fga, Hp*, and *Thbs1*) in NS, aged NS, BLM, and aged BLM groups, qRT-PCR verification was conducted. The results demonstrated that *Slc2a3* and *Fga* were significantly increased with age in both NS and BLM-stimulated mice (Figures 5A,B). Meanwhile, the *Hp* and *Thbs1* were found to significantly decreased with the increase of age (Figures 5C,D).

## Discussion

Aging is a strong risk factor and an independent prognostic biomarker for progressive IPF (22). However, a comprehensive analysis based on gene expression profiles for the investigation of the role played by aging in IPF was missing. In this study, using four human IPF datasets, we identified 108 altered aging-related DEGs in IPF. Similarly, through the analysis of a dataset of the BIPF mouse model, we identified 778 aging-related DEGs. Followed by that, both GO and KEGG analyses of the DEGs were conducted separately. The resulting DEGs were subsequently combined and generated eight common DEGs and four core genes. Finally, we confirmed that aging aggravated pulmonary fibrosis and validated the four aging-associated core genes in experimental pulmonary fibrosis.

In the present study, the 108 aging-associated DEGs of human IPF were found to be involved functionally in “response to glucocorticoid” and “response to corticosteroid” BP terms. Given the clinical practice at the time some of these samples were collected, it is likely some of these patients may have been receiving steroids, which could further influence this result. Although glucocorticoid showed no benefit to patients with IPF in several retrospective studies (23, 24), Zhang et al. implied that IPF with different gene types may benefit differently from glucocorticoid therapy (25). Previous research reported that dexamethasone treatments targeting the macrophages in long significantly alleviated pulmonary fibrosis through the regulation of the immune microenvironment (26). Generally, more comprehensive experimental designs might apply for exploring the role of glucocorticoids in IPF. Postsynaptic components are involved in most top five enriched CC terms, indicating an important role of the nerve system in aged patients with IPF. A longitudinal IPF cohort demonstrated that patients with IPF treated with α1 adrenoreceptor blockade displayed improvements in survival rates (27). Moreover, vagotomy attenuated symptoms of pulmonary fibrosis in BIPF mice by enhancing fibrogenic factors and fibrogenic cells (28). Growth factor activity was one significantly enriched MF term. Genes related to growth factor activity were downregulated in aged-related disease (29, 30). Nutrient metabolism imbalance was enriched in KEGG analysis. Loss of pyruvate kinase M2 resulted in cone degeneration in an age-dependent manner (31). Moreover, mitochondrial energy production reduction and cellular redox hemostasis loss played important roles in hearing loss associated with aging (32).

Different from DEGs of IPF, the significant GO enrichment of DEGs in the mouse model of BIPF was extracellular matrix (ECM) and collagen metabolism. Persistent ECM accumulation was considered to participate in the development of several diseases, including IPF, causing difficulties in reversing the disease progression of such disease (33). The ECM of aged lungs was featured by the upregulated transcriptional level of fibronectin extracellular domain A (Fn-EDA) and matrix metalloproteinases (MMPs), including MMP-2 and MMP-9 (5). Collagen contents were found higher in adult mice; however, its transcriptional level was found higher in young mice. This phenomenon was caused by dysregulations in collagen degradation (9). Despite the wide recognition of the association between ECM dysregulations and the process of aging, its underlying mechanisms were still unclear.

Age-associated DEGs of IPF and BIPF exhibited differences, while age-associated DEGs of HC and MC overlapped more. Inflammation and immunomodulation were enriched in age-associated DEGs of HC and MC. Age-related chronic inflammation is a major contributor to diseases with advancing age (34). Extensive evidence indicates that persistent low-grade inflammation is present in the aged population and that age-associated inflammation occurs in the lungs (35, 36). BALF collected from aged mice and aged humans contains increased levels of IL-6, TNF, and complement components, reflective of a disordered innate immunity (37). Although collagen and matrix synthesis are important in aged pulmonary fibrosis, the disordered inflammation and immune response cannot be ignored in aged pulmonary fibrosis (Figure 2B).

The GO terms of the common eight genes were enriched in the hyaluronan (HA) biosynthetic process. *Hp*, one of the four core genes, produced important effects in the process of lung injuries and repairments (38). Extracellular matrix Hp on type 2 alveolar epithelial cells (AEC2s) was critical for AEC2 renewal, repairments of lung injuries, and limitations of the development of fibrosis (39). *Thbs1* is associated with aging and promoted matrix homeostasis through the interaction with collagen, lysyl oxidase precursors, and sites of collagen cross-linking (40, 41). The relationship between *Thbs1* and aged pulmonary fibrosis was worth studying. *Slc2a3*, mediating the uptake of various monosaccharides, was reported to be associated with pulmonary fibrosis. Aging altered the uptakes of glucose and the transcription profile of *Slc2a3/Slc2a4* before and after spinal cord injury, which is potentially related to the inflammation level (42). *Fga* was cleaved by the protease thrombin to produce monomers. Subsequently, the monomers polymerized with fibrinogen beta (*Fgb*) and fibrinogen gamma (*Fgg*), forming the insoluble fibrin matrix. The level of *Fga* was higher in patients with IPF compared with that in healthy controls (43). Moreover, previous research found that the lack of a C-terminal fragment of *Fga* preceded the progress of fibrosis in patients with liver diseases (44). Most importantly, differences in the expression profiles of the four core genes were found in both patients with IPF and BIPF mice. In order to clarify that the expression changes of the four core genes were due to aging in the context of disease or aging alone, we analyzed the age-associated DEGs in HC and MC. Comprehensively, analysis of our research results, the bioinformatics analysis results, and the expression change of four core genes were due to aging in the context of pulmonary fibrosis.

Therefore, considering the crucial roles of these four core genes on the basis of this study and previous studies above, further studies may be focused on exploring the mechanisms under pulmonary fibrosis in the context of aging. There were still several limitations in the study. Firstly, we could not assess the role of other aging marks in pulmonary fibrosis, such as telomere attrition, epigenetic alterations, and genomic instability. They need more professional research methods, whereas we only explored the role of age in pulmonary fibrosis from the perspective of gene transcription. Secondly, the expression of the four core genes needs to be verified in humans. Thirdly, while our study was able to identify a number of novel pathways in aged IPF, it was limited by the available clinical data (such as the history of glucocorticoid use) and biases inherent in enrichment analyses. Fourthly, some gene information is lost in the process of data sets integration because it cannot be repeated in different datasets. Furthermore, more and larger-scale studies should be conducted.

It was widely recognized that aging was a dependent risk factor in pulmonary fibrosis. As shown by our study, aging-aggravated BLM induced pulmonary fibrosis in BIPF mice. The pathogenesis of young and aged pulmonary fibrosis was not exactly the same from the perspective of gene expression profiles. Four aging-associated core genes (*Slc2a3, Fga, Hp*, and *Thbs1*) were related to the development of pulmonary fibrosis.

## Data Availability Statement

The datasets presented in this study can be found in online repositories. The names of the repository/repositories and accession number(s) can be found in the article/Supplementary Material.

## Ethics Statement

The animal study was reviewed and approved by Animal Care and Use Committee of Tongji Hospital.

## Author Contributions

YL and JC: conceptualization, investigation, formal analysis, and writing original draft. SW: conceptualization, formal analysis, and writing original draft. ZT and MW: formal analysis. YF: formal analysis and writing original draft. JZ and JX: conceptualization and supervision. KT: conceptualization, investigation, and formal analysis. All authors contributed to the article and approved the submitted version.

## Funding

This study was supported by the National Natural Science Foundation of China (Nos. 81973986, 82170049, 82070032, and 81800041), Health Research Fund of Wuhan (No. WX21Q07), Health and family planning research project of Hubei (No. WJ2019M116), and the National Major Science and Technology Project for the Control and Prevention of Major Infectious Diseases of China (2017ZX10103004).

## Conflict of Interest

The authors declare that the research was conducted in the absence of any commercial or financial relationships that could be construed as a potential conflict of interest.

## Publisher's Note

All claims expressed in this article are solely those of the authors and do not necessarily represent those of their affiliated organizations, or those of the publisher, the editors and the reviewers. Any product that may be evaluated in this article, or claim that may be made by its manufacturer, is not guaranteed or endorsed by the publisher.

## Abbreviations

IPF: idiopathic pulmonary fibrosis
BLM: bleomycin
BIPF: bleomycin (BLM)-induced pulmonary fibrosis
GEO: gene expression omnibus
FC: fold change
DEGs: differently expressed genes
GO: gene ontology
KEGG: Kyoto Encyclopedia of Genes and Genomes
BP: biological process
CC: cell component
MF: molecular function
SPF: specific pathogen-free
NS: normal saline
BALF: bronchoalveolar lavage fluid
HE: hematoxylin-eosin
MT: masson trichrome
RT-PCR: real-time polymerase chain reaction
ANOVA: one-way analysis of variance
HC: healthy control
MC: mouse control
*Slc2a3*: solute carrier family 2 member 3
*Fga*: fibrinogen alpha chain
*Hp*: haptoglobin
*Thbs1*: thrombospondin 1
ECM: extracellular matrix
Fn-EDA: fibronectin extracellular domain A
MMPs: matrix metalloproteinases
AEC2: type 2 alveolar epithelial cells
*Fgb*: fibrinogen beta
*Fgg*: fibrinogen gamma.
