# Supplementary material for: Identification of Genetic Signature Associated With Aging in Pulmonary Fibrosis
Source: Front Med (Lausanne). 2021 Oct 20;8:744239. doi: 10.3389/fmed.2021.744239 (PMC8564051; doi:10.3389/fmed.2021.744239)
Supplement: Supplementary file 1 [file Table_1.DOCX]

Supplemental table 1. Primer sequence

| Gene |  | Sequence |
| --- | --- | --- |
| *Slc2a3* | Forward | ATGGGGACAACGAAGGTGAC |
| *Slc2a3* | Reverse | GTCTCAGGTGCATTGATGACTC |
| *Fga* | Forward | CACCTGCCTCATCTTGAGCG |
| *Fga* | Reverse | GCATTGACTCTGATGTCTCTCCA |
| *Hp* | Forward | GCTATGTGGAGCACTTGGTTC |
| *Hp* | Reverse | CACCCATTGCTTCTCGTCGTT |
| *Thbs1* | Forward | TTCTTACCCTTGACAACAACGTG |
| *Thbs1* | Reverse | CCACAGATAGCTTGGAGGTCC |
| *β-actin* | Forward | GGCTGTATTCCCCTCCATCG |
| *β-actin* | Reverse | CCAGTTGGTAACAATGCCATGT |

Supplemental Table 2. Characteristics of sample included.

|  | Young IPF | Aged IPF | P Value |
| --- | --- | --- | --- |
| Age | 47.79 (5.70) | 73.15 (2.71) | <0.001 |
| Gender |  |  | 1.000 |
| Female | 7/19 (36.8%) | 12/33 (36.4%) |  |
| Male | 12/19 (63.2%) | 21/33(63.6%) |  |

Abbreviations: IPF, idiopathic pulmonary fibrosis. The data are presented as the median (SD), n (%), or n/N (%). P values were calculated by the Mann-Whitney U test, χ² test.

**Supplemental Figure Legends**

**Supplemental Figure 1. DEGs associated with age in human HC.** (A) Volcano-plot of the 136 age-associated DEGs in HC. Pink circle: up-regulated genes with fold change over 1.5; Blue circles: down-regulated genes with a fold change over 1.5. (B) Top 10 GO biological processes analysis of the 136 age-associated DEGs. Outer gray circle: a scatter plot for each term of the logFC of the assigned genes; Red circles: up-regulation genes; Blue circles: down-regulation genes. (C) All GO cellular components analysis of 136 age-associated DEGs. (D) Top 10 GO molecular functions analysis of the 136 age-associated DEGs. (E) Top 15 significantly enriched KEGG pathways. DEGs, differently expressed genes; HC, healthy control; GO, Gene Ontology; FC, fold change; KEGG, Kyoto Encyclopedia of Genes and Genomes.

**Supplemental Figure 2. Age-associated DEGs in healthy control mice (MC)**. (A) Volcano-plot of the 1702 age-associated DEGs. Pink circle: up-regulated genes with fold change over 1.5; Blue circles: down-regulated genes with a fold change over 1.5. (B) Top 10 GO biological processes of the 1702 age-associated DEGs. Outer gray circle: a scatter plot for each term of the logFC of the assigned genes; Red circles: up-regulation genes; Blue circles: down-regulation genes. (C) Top 15 GO cellular components of the 1702 age-associated DEGs. (D) Top 15 GO molecular functions of the 1702 age-associated DEGs. (E) Top 15 significantly enriched KEGG pathways. DEGs, differently expressed genes; MC, mouse control; GO, Gene Ontology; FC, fold change; KEGG, Kyoto Encyclopedia of Genes and Genomes.

**Supplemental Figure 3. Common age-associated DEGs.** Venn diagram of DEGs of IPF patients, BIPF mice, HC patients and MC. IPF, idiopathic pulmonary fibrosis; BIPF, bleomycin induced pulmonary fibrosis; HC, healthy control; MC, mouse control.
