# Supplementary figures and images for: Identification of Genetic Signature Associated With Aging in Pulmonary Fibrosis
Source: Front Med (Lausanne). 2021 Oct 20;8:744239. doi: 10.3389/fmed.2021.744239 (PMC8564051; doi:10.3389/fmed.2021.744239)

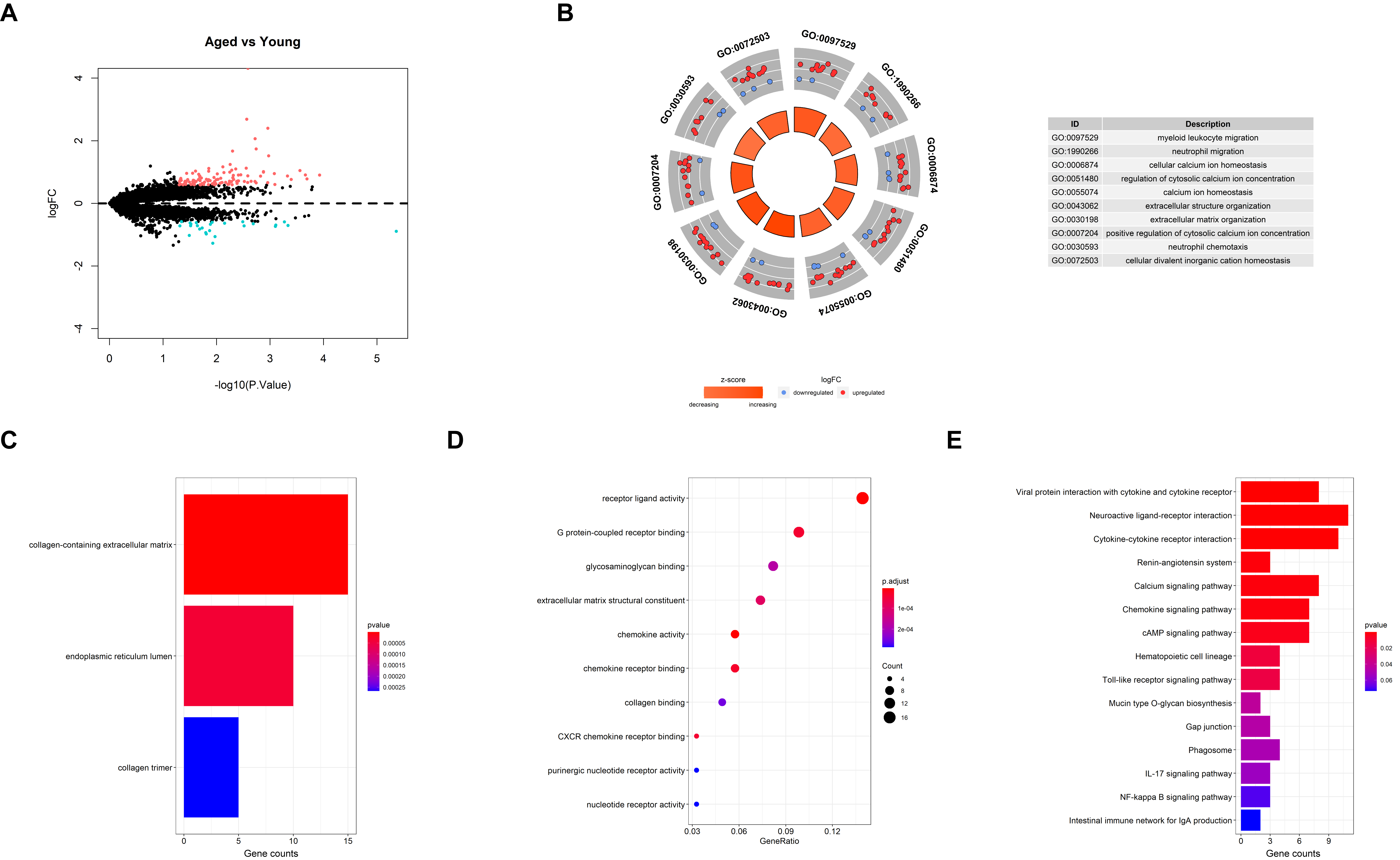

Supplement: Supplementary file 2 [file Image_1.TIF]

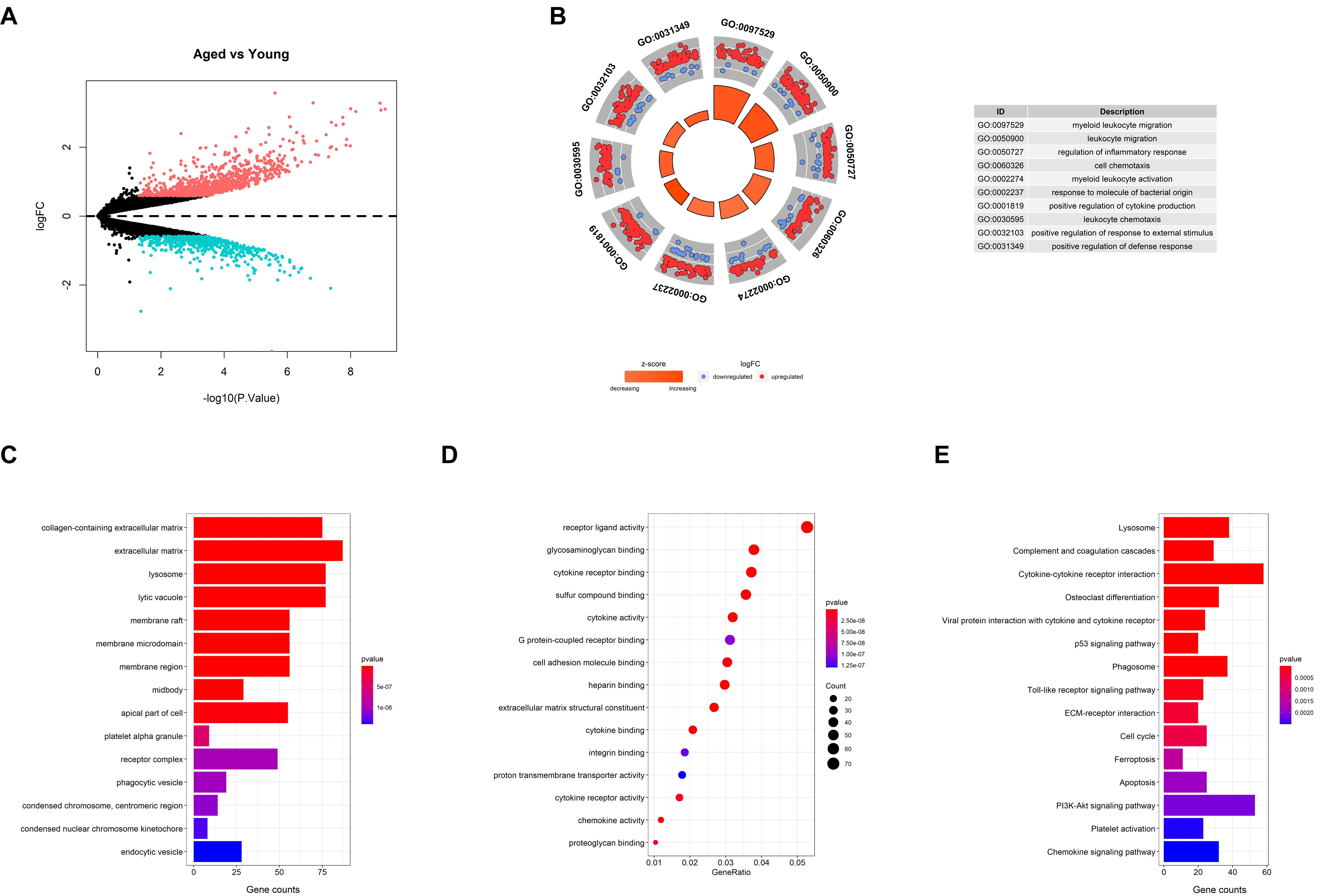

Supplement: Supplementary file 3 [file Image_2.TIF]

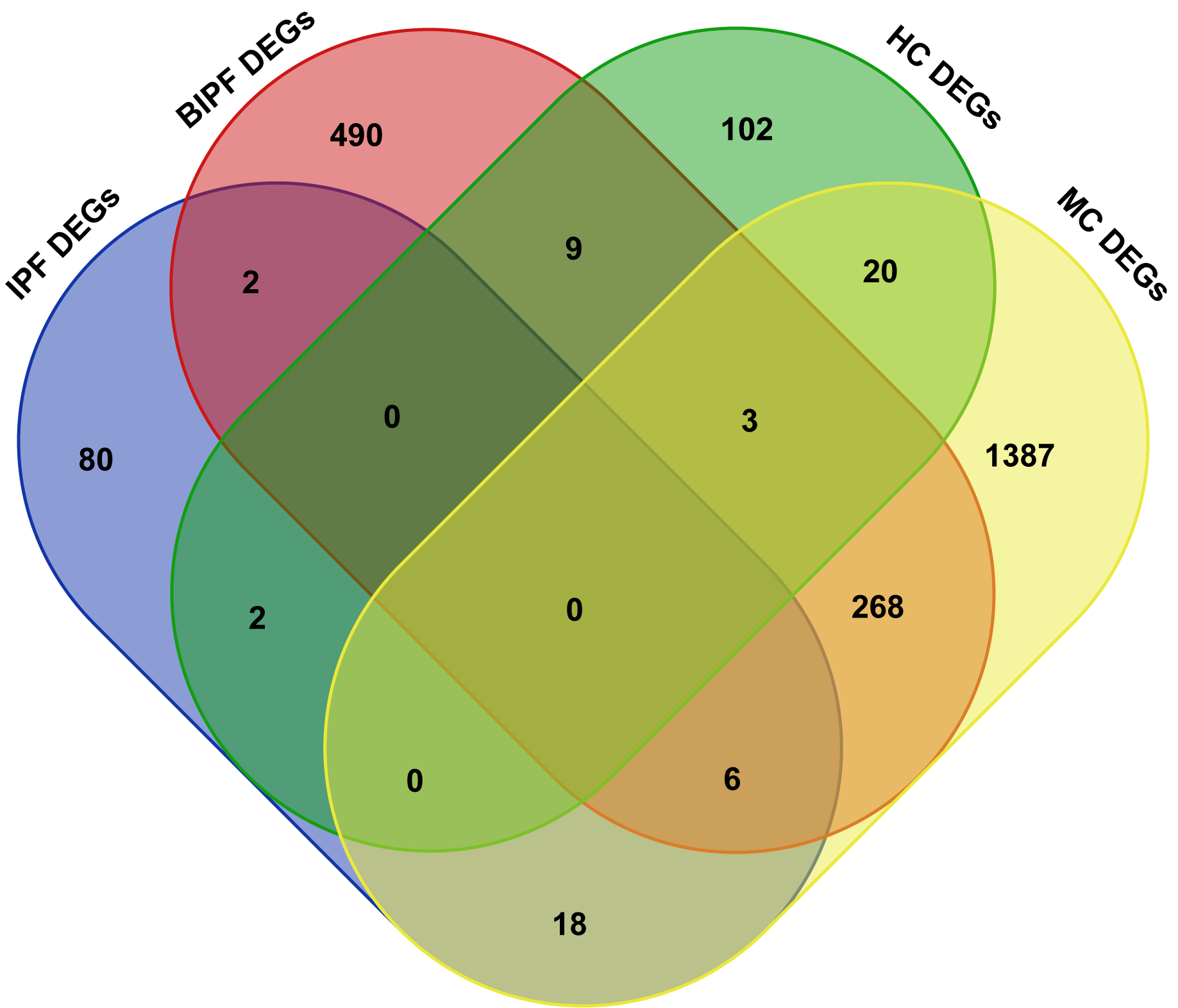

Supplement: Supplementary file 4 [file Image_3.TIF]
